# Emerging Evidence of Golgi Stress Signaling for Neuropathies

**DOI:** 10.3390/neurolint16020024

**Published:** 2024-03-07

**Authors:** Remina Shirai, Junji Yamauchi

**Affiliations:** Laboratory of Molecular Neurology, Tokyo University of Pharmacy and Life Sciences, Hachioji, Tokyo 192-0392, Japan; rshirai@toyaku.ac.jp

**Keywords:** Golgi stress, neuropathies, neuronal disease

## Abstract

The Golgi apparatus is an intracellular organelle that modifies cargo, which is transported extracellularly through the nucleus, endoplasmic reticulum, and plasma membrane in order. First, the general function of the Golgi is reviewed and, then, Golgi stress signaling is discussed. In addition to the six main Golgi signaling pathways, two pathways that have been increasingly reported in recent years are described in this review. The focus then shifts to neurological disorders, examining Golgi stress reported in major neurological disorders, such as Alzheimer’s disease, Parkinson’s disease, and Huntington’s disease. The review also encompasses findings related to other diseases, including hypomyelinating leukodystrophy, frontotemporal spectrum disorder/amyotrophic lateral sclerosis, microcephaly, Wilson’s disease, and prion disease. Most of these neurological disorders cause Golgi fragmentation and Golgi stress. As a result, strong signals may act to induce apoptosis.

## 1. Introduction

**Figure 1:**
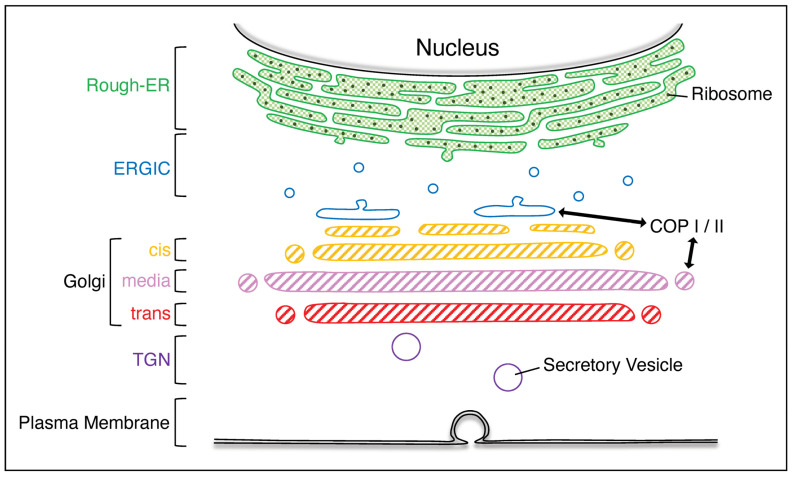
**Various organelles in mammalian eukaryotic cells.** The rough–ER is rich in ribosomes; the cargo produced from the rough–ER is transported via the ERGIC to the Golgi apparatus, which is composed of cis–, media–, and trans–Golgi. The cargo that has passed through the Golgi apparatus is transported out of the plasma membrane via the TGN. ERGIC, ER–Golgi intermediate compartment; TGN, trans–Golgi network.

Mammalian eukaryotic cells include various organelles, such as the nucleus, endoplasmic reticulum (ER), Golgi apparatus, lysosome, mitochondria, and peroxisome. Each organelle has a specific function. The Golgi apparatus plays a central role in intracellular protein transport by modifying a wide variety of proteins (cargo proteins) made in the ER with glycosylation and other modifications and transporting each to the designated organelle for functionality (Figure 1). The Golgi apparatus consists of stacks of flat cisternae, which collectively form structures known as Golgi ribbons. The Golgi stacks are polarized; the side of the stack that receives the cargo is called the cis–Golgi and the opposite side where the cargo leaves the stack is called the trans–Golgi. The enzymes that dispose of the cargo in the Golgi apparatus are widely distributed in a manner that reflects a sequential pattern of action on the cargo as it passes from cis– to trans–Golgi [1,2,3].

As cells undergo division, the organelles present in the cell other than the nucleus double in quantity and a mechanism exists to maintain the organelle quantity at a constant level. This is called the organelle quantitative regulation mechanism and it is essential for eukaryotes to function autonomously. When the quantitative regulatory mechanism of the ER collapses, an ER stress response occurs. Taniguchi et al. demonstrated that disruption of the Golgi quantitative regulatory mechanism causes the Golgi stress response and elucidated its molecular mechanism [4]. Thus, a certain enzyme is placed in the cis–Golgi, another certain enzyme in the trans–Golgi, and so on, in a given Golgi. Three main pathways of Golgi stress are known: the CAMP responsive element binding protein 3 (CREB3), transcription factor E3 (TFE3), and heat shock protein (HSP47) pathways; others are known to be involved in glycosylation [1].

Neurodegenerative diseases cause a gradual loss of neuronal cells in the brain or spinal cord, resulting in increased forgetfulness (dementia) or the inability to move the limbs properly (movement disorders). Golgi dysfunctions are often observed in neurodegenerative diseases, such as Alzheimer’s disease, Parkinson’s disease, Huntington’s disease, amyotrophic lateral sclerosis (ALS), and frontotemporal dementia (FTD) [5,6,7,8,9]. This paper provides an overview of Golgi stress, with particular reference to Golgi stress in neurodegenerative diseases.

## 2. The Role of the Golgi Apparatus

**Figure 2:**
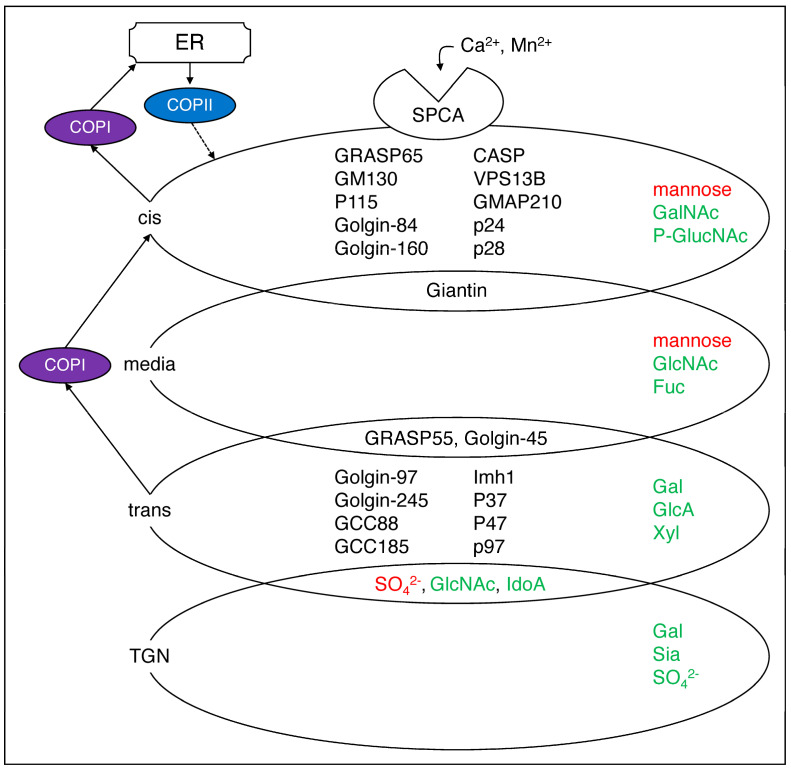
**Major components of the Golgi apparatus.** The Golgi cis, media, trans, or TGN have a specific sugar added at each point (in green) or removed (in red) [2]. In addition, the component proteins are distributed in the Golgi apparatus, some of them overlapping [3]. SPCA1 transports Ca^2+^ and Mn^2+^ into the Golgi secretion pathway while COP1 and COP2 are responsible for Golgi to vesicle and trans–Golgi to cis–Golgi reverse transport. The arrows are experimentally proven paths, while the dashed lines are expected paths. SPCA1, secretory pathway Ca^2+^–ATPase pump type 1; GRASP, Golgi reassembly and stacking protein; CASP, caspase; VPS13B, vacuolar protein sorting–associated protein 13B; GMAP, Golgi microtubule–associated protein; GCC, glutamate receptor–interacting protein and coiled–coil; GalNAc, N–acetylgalactosamine; GlucNAc, N–acetyl–d–glucosamine; Fuc, fucose; Gal; galactose; GlucA, glucuronic acid; Xyl, xylose; Sia, sialic acid; SO_4_^2−^, sulfate.

As mentioned in the previous section, the role of the Golgi apparatus is to modify the cargo transported from the nucleus through the ER and pass it to the plasma membrane and extracellular environment. The COP I and COP II coats are present around the Golgi apparatus. These vesicles are from the Golgi [10,11] or ER [12] membranes and often appear to have a ‘spiky appearance’ under cryo–electron microscopy (Figure 1 and Figure 2) [13]. COP I and COP II direct the transport of proteins across the membrane between the nascent compartments and play an important role in the selection of appropriate cargo proteins. COP I recycles proteins from the cis–Golgi apparatus to the ER while COP II selectively transports synthesized proteins from the ER to the Golgi apparatus [14]. The major enzymes present in the Golgi matrix have been largely elucidated and arranged in an ordered manner as a series of steps where the cargo is passed from the cis–Golgi to the media–Golgi and then to the trans–Golgi (Figure 2) [2,3,15,16,17]. Regularly arranged enzymes require metal ions to maintain the structure and function of the Golgi apparatus in order for it to be the Golgi apparatus [18]. Processing major enzymes in the Golgi apparatus lumen requires the presence of Ca^2+^ to function [19]. The Golgi apparatus contains elements such as Ca^2+^ pumps, Ca^2+^ channels, and Ca^2+^ binding proteins, which act as Ca^2+^ stores [20]. Cargo transported to the Golgi apparatus have sugars added by glycosyltransferases and sugars removed by glycosidases [2,21]. The cargo is proteolytically cleaved by proteases as it travels to the trans–Golgi network (TGN). These components are responsible for maintaining the unique structure and function of the Golgi apparatus. Among the components, Golgi reassembly and stacking proteins 65 (GRASP65) and GRASP55 play important roles in Golgi stacking, Golgi ribbon formation, cargo transport, cell cycle control, apoptosis, and autophagy [2,22,23,24]. Cytoplasmic dynein also has an important role in Golgi positioning and function [25]. Dynein, a motor protein, can move an organelle by moving it on microtubules. Dynein converts the chemical energy obtained from ATP hydrolysis into mechanical movement, forming giant Golgi ribbons [26].

## 3. The Golgi Stress Response

**Figure 3:**
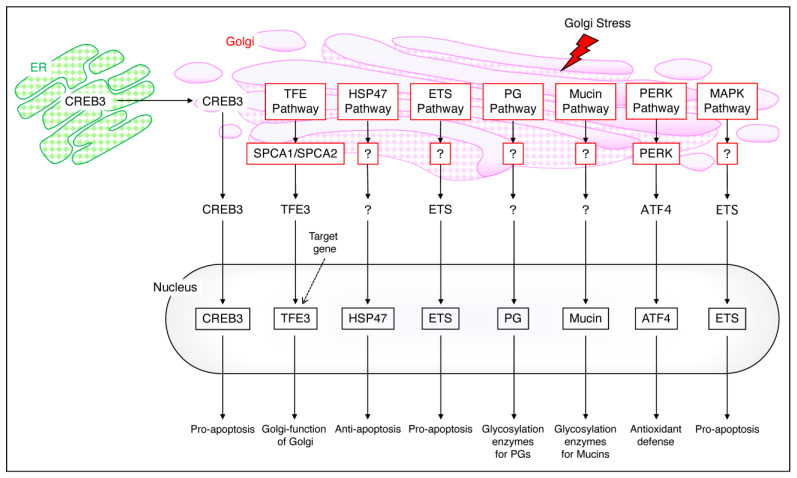
**The mammalian Golgi stress response.** The CREB pathway transports CREB3 from the ER to the Golgi and into the nucleus to cause pro–apoptosis. The TFE pathway maintains Golgi function via SPCA1/SPCA2 and TFE3, the HSP47 pathway causes anti–apoptosis, and the ETS pathway causes pro–apoptosis. PG migrates into the nucleus and undergoes glucosylation, and mucin also migrates into the nucleus and undergoes glucosylation; the PERK pathway mediates antioxidant defense via PERK and ATF4 and the MAPK pathway mediates pro–apoptosis via ETS. The arrows are experimentally proven paths, while the dashed lines are uncertain with expected paths.

When the function of the Golgi apparatus is impaired, the Golgi stress response is activated. Although the molecular mechanisms of the Golgi stress response remain largely unexplored, Figure 3 presents the six main pathways in mammals [20,27,28]. Each Golgi stress pathway is described below. 

### 3.1. The CREB3 Pathway

ER stress induces apoptosis; however, Golgi stress also induces apoptosis. Under Golgi stress, CREB3 is released from the ER to the Golgi, causing cell death by upregulating the transcription of ADP–ribosylation factor 4 (Arf4), which is required for the transport of cargo from the Golgi complex. Thus, there is a close relationship between the endoplasmic reticulum and the Golgi in the CREB pathway; when Golgi stress is induced, CREB3 serves as a sensor molecule in the ER stress response pathway [20].

### 3.2. The TFE Pathway

TFE3, which is involved in the TFE pathway, is involved in the structural maintenance and N–glycosylation of the Golgi apparatus. This pathway dephosphorylates TFE3 upon Golgi stress and transports it to the nucleus, where it activates the transcription of TFE3 target genes. Overexpression of the secretory pathway Ca^2+^–ATPase pump type 1 (SPCA1), which transports Ca^2+^ and Mn^2+^ into the Golgi secretory pathway, has been reported to cause Golgi swelling and promote nuclear translocation when TFE3 is dephosphorylated [29]. When the TFE pathway is activated, Golgi function itself is impaired.

### 3.3. The HSP47 Pathway

The HSP47 pathway, like the CREB3 pathway, is triggered by an increase in unmodified proteins in the Golgi. This pathway targets the HSP47 gene and inhibits apoptosis induced by the Golgi stress response; Nagata et al. used Benzyl 2–acetamido–2–deoxy–α–d–galactopyranoside (BG) to activate the HSP47 pathway. Two–dimensional electrophoresis identified HSP47 and showed that this endoplasmic reticulum chaperone is increased by heat stress, not by ER stress [30]. On the other hand, Miyata et al. reported that downregulation of HSP47 by siRNA causes cell death when o–glycosylation is inhibited and glycoprotein transport is blocked [31].

### 3.4. The ETS Pathway

The erythroblast transformation–specific (ETS) pathway is involved in the promotion of Golgi stress–induced apoptosis. The treatment of cells with Golgi stress inducers activates MEK1/2 and ERK1/2, which are upstream of the ETS pathway. Thus, the ETS pathway and the MAPK pathway are, in part, common; activation of the ETS pathway induces apoptosis; however, it is not associated with the endoplasmic reticulum, which is a major difference from the CREB3 pathway.

### 3.5. The PG and Mucin Pathway

Proteoglycans are glycoproteins and glycosylation of the PG is part of the function of the Golgi apparatus. Nadanaka et al. showed that the PG modified by glycosaminoglycan chains is involved in the regulation of a glycosyltransferase called EXTL2, which is involved in the synthesis of these glycans [32]. Mucin glycosylation is also part of the Golgi function and insufficient mucin glycosylation increases the transcriptional activity of mucin glycosyltransferases [20,27,28]. Mucin glycosylation occurs only in the Golgi; factors regulating the PG and mucin pathways remain unclear.

### 3.6. The PERK and MAPK Pathway

Activation of protein kinase R–like ER kinase (PERK), but not activated transcription factor 4 (ATF4), inhibits the translation of other proteins ATF4 induces and the production of CTH and hydrogen sulfide. Hydrogen sulfide increases intracellular cysteine levels and acts on antioxidant defenses [33]. The mitogen–activated protein kinase (MAPK) pathway, in commonality with the ETS pathway, induces apoptosis through Golgi stress via the ETS pathway.

## 4. Effect of Golgi Stress on Neuropathies

As mentioned above, Golgi stacking and Golgi ribbons are maintained by GRASP65 and GRASP55 while Golgi fragmentation is phosphorylated by kinases, such as Cdc2, GSK3β, RAF/MEK1/ERK1c, Plk1, Plk3, and other Golgi proteins, which, thereby, reversibly promote Golgi fragmentation. In contrast, Golgi fragmentation by apoptosis is irreversible and is proteolytically degraded via golgin, t–SNAREs, syntaxin 5, and GRASP–65 [34,35]. Nakagomi et al. found that GRASP–65 partially inhibited Golgi fragmentation by apoptosis. They also reported that transfection of the C–terminus of GRASP–65 inhibited Golgi fragmentation [36]. Treatment of HeLa cells with the ER stress inducer thapsigargin increases intracellular Ca^2+^ and protein kinase Ca (PKCa) activity and induces Golgi fragmentation by phosphorylating GRASP55 [24]. It has also been reported that inhibition of dynein leads to fragmentation of the Golgi apparatus in neuronal cells and that mutations in dynein and its regulators are associated with neurological disorders [25]. The processing and reassembly of fragmented Golgi bodies are reviewed by Shioi et al. [37]. Thus, Golgi morphology and neurodegeneration are closely linked; however, it is unclear whether the relationship between stress and the Golgi is involved in neurodegeneration. However, according to a report on the combination of bioinformatics modeling and literature mining conducted by Alvarez–Miranda et al., there is a clear bias between stress regulators and Golgi regulators in relation to neurodegeneration and it is, therefore, not clear whether stress is associated with neurodegeneration [38]. They declined to say that they were not “wet lab” experiments; however, they found that the factors identified as Golgi regulators are theoretically consistent with the processes involved in neurodegeneration. They add that they used HeLa cells rather than neurons and that there is room for further investigation of culture conditions.

**Figure 4:**
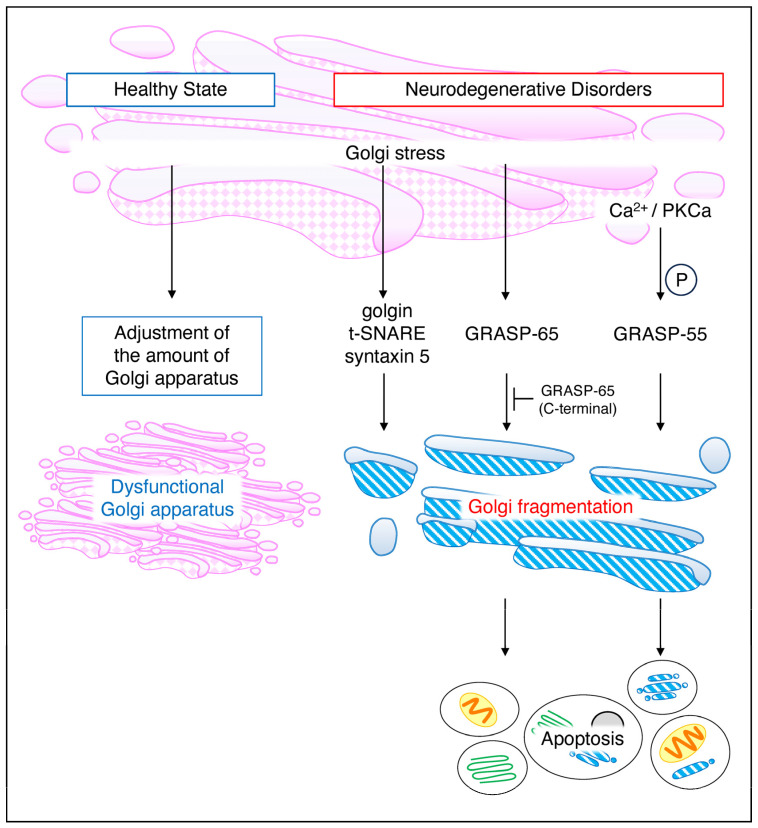
**Golgi stress in a healthy state and general neurodegenerative disorders.** Even under normal conditions, the Golgi stress response is always functioning, regulating Golgi volume in response to cellular demand. Deficient Golgi function results in an increase in intracellular Golgi volume. On the other hand, in neurodegenerative diseases, APP and BACE1 cause Golgi fragmentation via AP–1, AP–4, and Arf1/Arf4, a reversible response. Golgi–stress–induced golgin, t–SNARE, and syntaxin5 similarly cause Golgi fragmentation while GRASP–65 and GRASP–55 induce cell death. GRASP, Golgi reassembly and stacking proteins.

Although a review of Golgi stress occurring in neurodegenerative diseases, particularly Alzheimer’s disease, Huntington’s disease, and amyotrophic lateral sclerosis (ALS), has been reported by Fan et al. [39], this review has attempted to further broaden the scope of disease to cover neuropathies (Figure 4).

### 4.1. Alzheimer’s Disease and Parkinson’s Disease

Golgi fragmentation is known to occur in neurodegenerative diseases, such as Alzheimer’s disease and Parkinson’s disease. Golgi synthesizes and secretes amyloid precursor protein (APP) and beta–site APP cleaving enzyme 1 (BACE–1). APP transport is promoted in an AP–4–dependent manner and BACE–1 is promoted in an AP–1– and Arf1/Arf4–dependent manner [40]. The association between Caspase–3 and apoptosis is well known [41] and it has been reported that caspase–3 is involved in Golgi–stress–induced apoptosis [42]. Increased syntaxin 5 (Syx5) and βAPP in Alzheimer’s disease induces Golgi fragmentation and cell death is induced when Golgi stress becomes stronger and Caspase–3 is activated [43]. In addition, the induction of Golgi stress by monesin or nigericin in the hippocampus resulted in increased Syx5 levels and Golgi fragmentation while Syx1 and Syx6 levels were not affected, suggesting that Syx5 may mediate the Golgi stress response [44,45]. Additionally, α–Synuclein, which is characteristic of Lewy body dementia, induces transcriptional regulation. Paiva et al. reported that mutant forms of α–Synuclein promote transcriptional regulation, increase DNA binding, cause Golgi fragmentation and ER stress in dopaminergic spermatocytes, and also increase sensitivity [7].

Aβ, the putative causative protein of Alzheimer’s disease, is converted to insoluble Aβ fibrils upon accumulation; when Aβ fibrils accumulate in neurons, they exhibit the most neurotoxic effects. In 2005, Lee et al. reported that HSP20 suppressed the accumulation of insoluble Aβ fibrils in SH–SY5Y and PC12 neuronal cells [46]. HSP20 is neuroprotective but fails to exert this effect when serine 16 phosphorylation is inhibited. Lu et al. performed oxygen–glucose deprivation and reperfusion (OGD/R) of neuroblastoma cells and showed that the transfection of HSP20 mutants resulted in Golgi bodies swelling and fragmenting [47]. Nelson et al. reported that Stimulator of Interferon Gene (STING) –interferon signaling was impaired in Alzheimer’s disease and interferon expression was not induced because transport from the Golgi to the ER did not occur [8]. The increased production of Aβ or neurofibrillary tangles (NETs) in Alzheimer’s disease has been shown to lead to Golgi fragmentation, creating a self–perpetuating cycle [48]. Among Alzheimer’s disease, most cases of early familial Alzheimer’s disease are associated with Presenilin–1 (PS1)/PS2 mutations. Familial Alzheimer’s–disease–associated PS1 variants reduce the unfolded–protein response (UPR) in the ER by the proteolysis of APP, which induces ER stress. Furthermore, PS1 has been reported to inhibit ER–Golgi–stress–induced apoptosis as the expression of PS1 mutant forms correlates with the induction of apoptosis [49]. With regard to PS1, Ueda et al. also mentioned the endocytic pathway in relation to PS1: they reported that when each transport pathway of endosomes was individually inhibited in neuroblastoma cells, PS1 was retrieved from endosomes to the Golgi apparatus only when endocytosis was impaired and, then, further transported to the endoplasmic reticulum for degradation by proteasomes [50].

**Figure 5:**
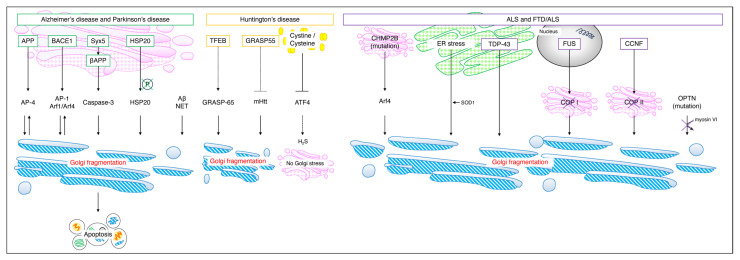
**Golgi stress in each neuropathy.** In Alzheimer’s disease and Parkinson’s disease, Syx5 induces Golgi fragmentation and cell death via βAPP and Caspase–3. The inhibition of HSP20 phosphorylation and accumulation of Aβ and NETs cause Golgi fragmentation. It has been reported that in Huntington’s disease, increased intracellular cystine/cysteine inhibits ATF4 and suppresses hydrogen sulfide production so that Golgi stress does not occur; however, there are scattered reports to the contrary [33,55]. In FTD/ALS, the accumulation of CHMP2B mutants in the Golgi activates Arf4 and causes Golgi fragmentation. The accumulation of TDP–43 in SOD1 and the ER and changes in the localization of COP I and COP II by FUS and CCNF also cause Golgi fragmentation. APP, amyloid precursor protein; BACE1, beta–site APP cleaving enzyme 1; NET, neurofibrillary tangle; mHtt, mutant huntingtin; CHMP2B, charged multivesicular body protein 2B; SOD, superoxide dismutase 1; TDP–43, TAR DNA binding protein 43; FUS, fused in sarcoma; CCNF, Cyclin F; OPTN, optineurin gene.

Next, we will outline the relationship between Golgi stress and substances that have recently been reported as candidates for the treatment of Alzheimer’s disease. Golgi brefeldin A resistance factor 1 (GBF1), an Arf–guanine nucleotide exchange factor (Arf–GEF), initiates Arf at the trans–Golgi and plays an important role at the ER–Golgi interface. The inhibition of GBF1 by Brefeldin A also inhibited APP transport, resulting in ER stress. This suggests that GBF1 may be a potential therapeutic target for Alzheimer’s disease [51]. Also, Salvianolic acid B is a bioactive substance isolated from Salvia miltiorrhiza, a Chinese herbal medicine with potential for the treatment of neurodegenerative disorders, such as Alzheimer’s disease and Parkinson’s disease. The effect of Salvianolic acid B on retinopathy, which occurs earlier than cognitive impairment in early Alzheimer’s disease, was examined by Wang et al. They showed that in the neuronal SH–SY5Y cells, Salvianolic acid B reduced Aβ production by decreasing BACE1 expression and inhibiting its transport to the Golgi [52]. In addition, there have been recent reports on metal ions and neuroprotection. The heavy metal zinc is most abundant in the brain and is essential for the maintenance of normal brain function and brain development [53]. Zinc homeostasis is regulated by zinc transporter families (ZnTs) and ZnT7 is present in the ER and Golgi apparatus. Lee et al. found that ZnT7 is important for neurodevelopment and that the knockdown of ZnT7 did not alter Aβ deposition but worsened neurodegeneration. These results suggest that proper distribution of zinc in the Golgi may be useful in protecting neurons and Alzheimer’s disease [54] (Figure 5).

### 4.2. Huntington’s Disease

Huntington’s disease is a polyglutamine disease caused by mutations in the huntingtin gene and involves abnormal repeats in the glutamine–encoding CAG. GCP60 (also called acyl–CoA binding domain containing 3, or ACBD3) levels have also been shown to be elevated in Huntington’s disease due to an expanded polyglutamine repeat sequence in huntingtin. Mutant huntingtin (mHtt), a striatal protein ras homolog enriched in striatum (Rhes), binds to GCP60 and causes apoptosis [56].

Ahat et al. showed that mHtt is secreted in a GRASP55–dependent manner, which is implicated in most neurodegenerative diseases. They reported the autophagy–dependent enhancement of the unconventional secretion of Htt under stress conditions. The inhibition of mHtt secretion by GRASP–55 knockout also enhanced Htt aggregation and toxicity [57]. Since GRASP–55 plays an important role in maintaining the Golgi structure, GRASP–55 knockout resulted in Golgi fragmentation. Abnormalities of Hdh due to glutamine chain extension in Huntington’s disease were reported using a battlefield somatic cell line established from Hdh (Q111) knock–in embryos; Hdh (Q111) showed a phenotype distinct from huntingtin loss and overexpression, similar to the full–length mutant protein. The results indicate that specific pathways, such as increased p53, ER stress, Golgi stress, and hypoxia, are involved in Huntington’s disease [58]. Secretory carrier membrane protein 5 (SCAMP5) is a membrane protein induced in the brains of Huntington’s disease patients and its expression is regulated by transcription factor EB (TFEB), a transcriptional regulator of autophagy. SCAMP5 inhibits autophagy by blocking autophagosome–lysosome fusion but does not cause intracellular protein aggregation. Compensatory induction of Golgi fragmentation and stimulation of α–Synuclein secretion indicates that SCAMP5 exits proteins via exosomes, not via the autophagy–lysosome pathway [59].

On the other hand, it is known that intracellular cystine and cysteine are increased in Huntington’s disease; however, it has been reported that these do not induce Golgi stress. In Huntington’s disease, which is involved in the PERK pathway, the induction of ATF4 is inhibited by the expression of cystine and cysteine, hydrogen sulphide expression is reduced, and Golgi stress does not occur. However, treatment with monensin, which induces Golgi stress, increases cysteine and hydrogen sulphide levels via the PERK/ATF4 pathway and protects cells [33,60,61]. In addition, in striatal neuronal loss during Huntington’s disease, the transient receptor potential canonical type 5 (TRPC5) is activated by oxidative stress. The specificity of Cys–181 for TRPC5 function is high and the inhibition of Cys–181 palmitoylation destabilizes TRPC5 tetramer formation. Conversely, when appropriate S–palmitoylation occurs at Cys–181, TRPC5 assembly is transported from the ER to the plasma membrane via the Golgi apparatus and stabilized [55] (Figure 5).

At present, there is no curative therapy for Huntington’s disease. Subodio et al. demonstrated that Golgi–stress–inducing monensin acts via the PERK/ATF4 pathway and that monensin activates the PERK/ATF4 pathway, thereby restoring the cysteine biosynthetic enzyme cystathionine γ–lyase (CSE). Subodio et al. restored cystathionine γ–lyase (CSE), a cysteine biosynthetic enzyme, by activating the PERK/ATF4 pathway with monensin [61]. This suggests that restoring CSE function may be useful in the treatment of other diseases involving Golgi stress via the PERK/ATF4 pathway [28].

### 4.3. ALS/FTD

ALS is a disease of unknown cause in which motor neurons in the brainstem and spinal cord degenerate or disappear, weakening the muscles necessary to maintain vital activity. FTD is a disease in which atrophy of the frontal and temporal lobes causes language impairment but memory is generally preserved. Both diseases present different symptoms but some of the causative genes are on a single spectrum. ALS is partly due to Golgi fragmentation, as well as the misfolding of superoxide dismutase (SOD1); however, Golgi fragmentation is thought to be caused by pathogenic mutant proteins that inhibit vesicular transport between the endoplasmic reticulum and the Golgi apparatus and between the Golgi and the plasma membrane [62]. It is considered to be caused by pathogenic mutant proteins that inhibit ER–Golgi and Golgi–to–plasma membrane vesicle trafficking [63]. This results in the dysfunction of autophagy and axon homeostasis. Proteins such as superoxide dismutase 1 (SOD1), TAR DNA binding protein 43 (TDP–43), and fused in sarcoma (FUS) are associated with neurodegeneration in ALS and Soo et al. reported that mutations in these genes inhibit protein transport in neurons. ER–Golgi transport was process–dependent but there was commonly a dependence on Rab1. We recently reported that the charged multivesicular body protein 2B (CHMP2B) mutation in the FTD/ALS–type 7–related gene inhibits neurite outgrowth; the CHMP2B mutant accumulates mutant protein in the Golgi and activates the Golgi stress signal Arf4. Additionally, the siRNA–based knockdown of Arf4 restored the neurite outgrowth inhibited by the CHMP2B mutant, suggesting that the CHMP2B mutant inhibits neuronal morphogenesis by inducing Golgi stress signaling [5]. Misfolding and aggregation of SOD1 is one of the hallmarks of ALS; however, it has been unclear whether the intracellular uptake of SOD1 is the initiator of neurodegeneration. Also, the presence of Rab1–positive inclusions in ALS patients also suggested that secretory protein transport, the transport of secreted proteins, may be inhibited [64]. The G4C2 hexanucleotide repeat of C9orf72 is the most common cause of FTD/ALS. G4C2 insertion lengths vary; however, arginine–rich dipeptide repeat protein (DPR) translated from G4C2 is more toxic. Proteomic analysis of the Drosophila brain by Oset et al. showed that the brain proteome is altered in a repeat length–dependent manner and that expression of poly–glycine–alanine (GA) upregulates proteins involved in ER to Golgi trafficking. Downregulation of Tango1, which regulates the ER to Golgi transport, restored GA400 toxicity, suggesting that the misregulation of GA400 contributes to poly–GA toxicity [6]. TDP–43 accumulates in the cytoplasm of neurons and glial cells in the brain of ALS patients and also causes FTD/ALS by associating with stress granules formed in response to endoplasmic reticulum stress and other stresses to form large inclusion bodies. Inclusion bodies of the C–terminal fragment of TDP–43 are formed by association with the endoplasmic reticulum and Golgi compartments, resulting in ER dysfunction [65]. In other words, it causes Golgi stress via the endoplasmic reticulum.

Many reports indicate that ALS/FTD results in impaired vesicle–Golgi trafficking and Golgi stress. First, we present a paper on RNA metabolism. To identify Golgi–stress–induced secreted proteins, Baron et al. developed a quantitative proteomics approach called BONLAC, combining biorthogonal noncanonical amino acid tagging (BONCAT) and stable isotope labeling by amino acids in culture (SILAC) to detect newly synthesized ultra–trace proteins, a quantitative proteomics approach called BONLAC. As a result, hundreds of proteins were identified by active translation of the proteome under arsenite stress conditions. Among them, the identified FUS is a RNA–binding protein, a molecule that is primarily found in the nucleus and functions in all aspects of RNA metabolism, including transcription, selective splicing, and RNA transport. In primary and human–stem–cell–derived neurons expressing FUS mutants, COPB I localization was altered and COPB I and Golgi signaling was dispersed [66]. In other words, transport from the endoplasmic reticulum to the Golgi was impaired. Next, we will discuss metabolism by SOD. The misfolding and aggregation of zinc–coordinating SOD1, a reactive oxygenase, is one of the hallmarks of ALS; however, it was unclear whether the intracellular uptake of SOD1 is an initiator of neurodegeneration [67,68]. Sundaramoorthyet al. showed that extracellular wild–type and mutant SOD1 is internalized into cells by macropinocytosis and is independent of SOD1 mutation or misfolding. They further reported that the SOD1 mutant added exogenously to the neuronal SH–SY5Y or NSC–34 cells, which resulted in Golgi fragmentation due to the disruption of ER–Golgi protein transport [69]. ER–Golgi transport was also inhibited in embryonic motor and cortical neurons from SOD1^G93A^ mice, a widely used mouse model of ALS. Finally, abnormalities associated with secretory vesicles are outlined. In the Rab family, which is important for secretory proteins, the presence of Rab1–positive inclusions in ALS patients also suggests that the transport of secretory proteins may be impaired [64]. Williams et al. newly identified missense mutations in Cyclin F (CCNF) [NM_001761.3] (also called FBXO1) as a cause of familial FTD/ALS [70]. In experiments with cortical primary neurons, Ragagnin et al. reported that CCNF encoding Cyclin F inhibits the transport of secreted proteins from the ER to the Golgi by a mechanism involving COPII dysregulation [71]. Fifita et al. analyzed whole exosome sequence data from 74 ALS patients and identified a novel mutation in the optineurin gene (OPTN) that is found in less than 1% of FTD/ALS patients: expression of the OPTN mutant in motor–neuron–like NSC–34 cells resulted in Golgi fragmentation and a novel heterozygous missense mutation in OPTN (c.883G > T, p.Val295Phe) also caused Golgi fragmentation [72]. The OPTN also functions as an adaptor protein linking the molecular motor myosin VI to secretory vesicles and autophagosomes. The mutations p.Q398X and p.E478G disrupted the binding between OPTN and myosin VI, causing the inhibition of secretory protein transport and fragmentation of Golgi bodies [73] (Figure 5).

### 4.4. Other Neuropathies

Mutations in the gene encoding aminoacyl–tRNA–synthase–complex–interacting multifunctional protein 2 (AIMP2) are responsible for hypomyelinating leukodystrophy 17 (HLD17), a condition characterized by myelin dysplasia and oligodendrocyte shedding. These mutations result in the aggregation of the AIMP2 protein and its localization to the Golgi apparatus. Notably, AIMP2 mutant forms are linked to Caspase–2 activation. This aberrant activation of Caspase–2 is associated with upregulated Golgi stress in individuals with AIMP2 mutations. The knockdown of Caspase–2 reversed the wild–type and mutant phenotypes, suggesting that AIMP2 stimulates Caspase–2 in response to Golgi stress and suppresses oligodendrocyte differentiation [74]. HDL type 1 (also known as Pelizaeus–Merzbacher disease) is caused by missense mutations or duplications in the proteolipid protein 1 (PLP1) gene. Numata et al. found that intracellular expression of the PLP1–A243V mutant, a severe cause of HLD1, reduced ER chaperones with KDEL (Lys–Asp–Glu–Leu). The mutant also causes Golgi fragmentation; however, the mild PLP1 mutant shows little organelle change. Furthermore, the PLP1 mutant reduces the localization of the KDEL receptor, which transports KDEL chaperones back from the Golgi to the ER, suggesting that Golgi fragmentation may be associated with the severity of HLD1 [75].

Congenital microcephaly affects neuronal commitment and survival and is associated with intellectual disability. As the ER and Golgi maintain cellular homeostasis through proteostatic regulation, Golgi stress is thought to induce microcephaly. Passemard et al. reported that TFE3, ETS, and CREB are induced during Golgi stress, inducing Golgi structure and pro–apoptotic factors [76]. 

Wilson’s disease, a copper metabolism disorder, presents with Parkinsonian–like extrapyramidal symptoms and is characterized by changes in the basal ganglia. The APP is a copper–binding protein and copper is the main source of free radical formation in the brain. To prevent such oxidative stress, the APP has a copper–binding domain, which acts as a metal transporter in neurons, and it is predicted that Wilson’s disease is associated with dopaminergic disorders [77].

Prion diseases cause neurodegeneration due to misfolding of the prion protein and Puig et al. have shown that deletion of the C–terminal domain of the prion protein leads to neuronal shedding and p38–MAPK activation in the hippocampus and cerebellum and inhibits the Golgi–cell–membrane secretion pathway [78]. Bouybayoune et al. also reported that transgenic mice with a C–terminal mutation in the prion protein had inhibited transport from the Golgi [79]. So, is Golgi stress involved in hyperglycemia–induced neuroinflammation in diabetes neuroinflammation, which is a complication in many diabetes patients? Li et al. reported on this question. They found that in BV2 cells, high glucose caused Golgi fragmentation and enhanced the interaction between Vacuolar protein sorting ortholog 35 (VPS35) and Golph3. They also reported that high glucose regulates Golgi stress via the NLR family pyrin–domain–containing 3 (NLRP3)/VPS35/Golph3/Vimentin pathway [80].

## 5. Conclusions

In this review, the role of the Golgi was first outlined. Although several signaling pathways have been identified for Golgi stress, the transmission from the Golgi to the nucleus remains unclear. Conversely, the PERK and MAPK pathways, which are strongly involved in cellular maintenance as well as Golgi stress, are well understood. However, whether the elevation of these Golgi stress markers is a cause or a consequence of neurodegenerative disease remains a matter of debate. In terms of neurological disorders, Golgi stress in Alzheimer’s disease, Parkinson’s disease, and Huntington’s disease has been explored. Although Alzheimer’s disease and Parkinson’s disease share common stress signals, the Golgi stress in Huntington’s disease appears to follow distinct pathways from the other two disorders. Although Golgi stress has been extensively studied in FTD/ALS, the pathway of ER–stress–induced Golgi stress is still largely unexplored. On the other hand, there were scattered reports of an accumulation of mutagens in the Golgi causing Golgi fragmentation. Other diseases described include HLD, microcephaly, Wilson’s disease, prion disease, and hyperglycaemia–induced neuroinflammation. All of these diseases cause Golgi stress and the presence of stress signals in common with diseases with very similar pathologies suggests the possibility of major Golgi–stress–inducing factors. In addition, there have been only a few reports on neurodegenerative diseases and Golgi stress in vivo and further research is needed. While signaling pathways involved in Golgi stress have been reported, intervention for diseases that involve Golgi abnormalities and lack treatment is an urgent issue.
